# Measuring electrophysiological changes induced by sub-concussive impacts due to soccer ball heading

**DOI:** 10.3389/fneur.2025.1500796

**Published:** 2025-03-06

**Authors:** Geoffrey Brookshire, Angelo Pennati, Keith J. Yoder, MacKenzie Tweardy, Colin Quirk, Marilyn Perkins, Spencer Gerrol, Steven Raethel, Devin Nikjou, Simona Nikolova, Michael Leonard, Amy Crepeau, David W. Dodick, Todd J. Schwedt, Ché Lucero

**Affiliations:** ^1^SPARK Neuro Inc., New York, NY, United States; ^2^Special Operations Command Central (SOCCENT), Tampa, FL, United States; ^3^Department of Neurology, Mayo Clinic, Phoenix, AZ, United States; ^4^School of Biological and Health Systems Engineering, Arizona State University, Tempe, AZ, United States; ^5^Atria Academy of Science and Medicine, New York, NY, United States

**Keywords:** electroencephalography, traumatic brain injury, concussion, sub-concussive impact, repetitive head impacts, sports injury

## Abstract

A growing body of research suggests that impacts to the head, including sub-concussive impacts, carry risks for long-term detrimental effects on cognition and brain health. Despite the potential for negative health consequences associated with sub-concussive impacts, there is currently no reliable and objective method used in clinical practice to assess whether a particular sub-concussive impact affected the brain. In this preliminary study, we developed a machine-learning classifier to detect changes in brain electrophysiological activity following sub-concussive impacts that occur during soccer ball heading. We recorded EEG from soccer players before and after they repeatedly headed a soccer ball, and trained classifiers to distinguish between an individual's EEG patterns before and after these sub-concussive impacts. The classifiers were able to identify post-impact EEG recordings with significantly higher accuracy than would be expected by chance, both 1 h and 24 h after the impacts occurred. After controlling for electrophysiological changes attributed to exercise, changes to brain activity attributable to soccer heading were detectable at 24 h post-heading, but not at 1-h post-heading. The observed time-course of EEG changes mirrors a similar pattern seen in traumatic brain injury, in which an inflammatory cascade is manifest 24 to 48-h post-injury; we suggest that EEG changes following sub-concussive impacts may stem from inflammation or some other physiological process that unfolds on a similar timescale. These results are an important step toward developing an EEG-based tool that can assess whether electrophysiological consequences are present following sub-concussive head impacts.

## 1 Introduction

Traumatic brain injury (TBI) has been dubbed a “silent epidemic” (1), leading to 50,000 deaths and 80,000 permanent disabilities in the United States every year (2). While it's clear that TBIs pose a serious public health concern, less is known about the risks and effects of sub-concussive impacts—those that do not trigger clinical symptoms of concussion or TBI (3). There is not currently any widely used test available to detect the potential consequences of sub-concussive impacts on brain electrophysiology. With the goal of advancing the medical community's ability to accurately study and identify potential consequences of sub-concussive impacts, we investigated the use of electroencephalography (EEG) to detect changes in brain activity associated with soccer ball heading.

TBI triggers a cascade of neurological damage mediated by changes in cerebral blood flow, cerebral metabolic dysfunction, neuroinflammation, blood-brain barrier disruption, and impairment of cerebrovascular autoregulation (4–6). TBI damage has shown to be cumulative, such that repeated TBIs can lead to decreased neuropsychological performance (7) and adverse cellular changes (8, 9). These effects are particularly evident in retired American football players with a history of multiple mild TBIs (mTBIs), who exhibit increased rates of cognitive impairment (10) and greater incidence of chronic traumatic encephalopathy (CTE) (11–13). A history of repeated head impacts has also been found to correspond to cumulative neurological damage in boxing (14, 15), ice hockey (12, 16), rugby (17), and soccer (18, 19).

Recent studies suggest that repetitive head impacts may be a cause for concern even when they do not result in symptoms of concussion (20, 21). Sub-concussive impacts do not trigger the same immediate outward signs of neurological dysfunction as mTBI, but they may cause microstructural and functional changes in the brain (3, 20). The effects of sub-concussive impacts might accumulate over time, leading to neurological impairment and lowering the threshold for sustaining future TBIs (22).

Athletes can experience several hundred to more than a thousand sub-concussive impacts each year (22). Even when no symptoms are present, effects of sub-concussive impacts may be apparent in neuropsychological testing, structural and functional brain imaging, and autopsy; these methods reveal signs of brain damage in individuals who had no history of concussion, but who were engaged in contact sports or exposed to repeated explosive blasts during military service (22). Furthermore, PET scans reveal accumulation of amyloid proteins in otherwise healthy military instructors who were exposed to repeated sub-concussive blasts (23).

Despite the potential risks associated with sub-concussive impacts, the condition remains ill-defined, and there is a lack of reliable, sensitive, and specific measures to determine whether a sub-concussive head impact affected the brain. Standard assessments for TBI often rely on subjective measures and are prone to under-reporting (24), and their long-term usefulness is questionable (25). Furthermore, these assessments for TBI might not be sensitive enough to detect sub-concussive impacts that cause changes in brain electrophysiology without causing overt symptoms (24). Also, standard clinical assessments for TBI are typically limited by requiring a baseline measurement to be acquired prior to the injury (26).

EEG has emerged as a potential diagnostic tool for several different neurological disorders beyond epilepsy (27). For example, researchers have developed EEG classifiers to detect and grade the severity of dementia (28–30). TBIs have been associated with a variety of changes in EEG activity, including increased alpha-band (7.5–10 Hz) power and decreased theta-band (3.5–7.5 Hz) power (31). Sub-concussive impacts have also been associated with changes in EEG activity (32–34). For instance, spectral features of EEG depend on the number of sub-concussive impacts experienced during a kickboxing match (35), and a variety of event-related potential (ERP) components predict the number of sub-concussive impacts experienced by amateur hockey players (32). Based on these existing data, the objective of this study was to determine if an accurate classification model for sub-concussive impacts could be built using resting EEG data.

In the present study, we recorded EEG from soccer players before and after they experienced sub-concussive impacts by heading a soccer ball. We then trained a classifier to distinguish between an individual's EEG patterns before and after these sub-concussive impacts. We hypothesize that EEG signals contain information that can be used to identify whether participants have experienced sub-concussive impacts. This technology represents a preliminary step toward improved abilities to identify brain electrophysiological consequences that may result from sub-concussive impacts.

## 2 Materials and methods

We recorded resting EEG before, 1 h after, and 24 h after participants performed a ball-passing task using their head (i.e., repeatedly headed a soccer ball). We then tested whether machine learning classifiers could identify a given recording as having occurred before or after the sub-concussive impacts.

### 2.1 Participants

We recruited participants who had at least 1 year of experience playing soccer and considered themselves to be active players (*N* = 36; 22 males and 14 females; mean age 28.2 years, median age 28 years, range 19–38). Participants were non-smokers and reported no prior history of TBI in the preceding 12 months, and no history of vestibular, ocular, or vision dysfunction. Participants were asked to refrain from using recreational drugs or alcohol for 24 h before each study session, and were instructed to not engage in any activities outside of the study that may result in head impacts.

Participants were recruited using flyers and online postings at the Mayo Clinic in Arizona and the surrounding community. Recruitment flyers were shared with two local adult community soccer leagues. Participants were compensated $200 upon completing the study.

The study was approved by the Mayo Clinic Institutional Review Board and each participant completed an informed consent process including signing informed consent forms. The study was pre-registered at clinicaltrials.gov (NCT05562544).

### 2.2 Experimental procedures

**Figure 1:**
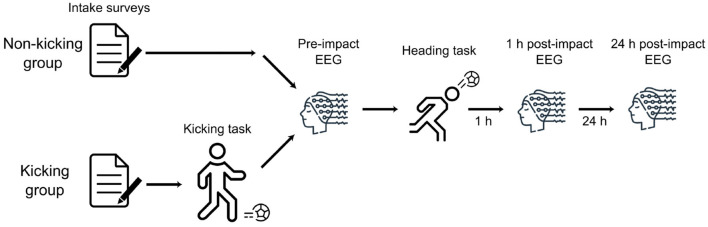
Experimental procedures: Participants began by providing informed consent and completing intake surveys. Those participants who had been assigned to the “kicking” group then performed a passing task by kicking the soccer ball. All subjects then performed the same procedures from that point on, starting with an EEG measurement session (pre-heading EEG). Next, participants performed a passing task by heading a soccer ball. After waiting 1 h, participants performed a second EEG measurement session. Approximately 24 h later, participants returned to the lab to perform a final EEG measurement session.

Half of the participants (*n* = 18) began by performing a ball-passing task with their feet (“kicking task”; Figure 1). The remaining participants began the study without performing the kicking task. Participants were randomized into one of the two groups by alternating the group that each successive subject was assigned to. From this stage on, the experimental procedures were identical for both groups of participants.

All participants underwent a measurement session prior to performing the soccer ball heading task. The measurement session included resting-state EEG, the King Devick Test (KDT), and an eye-tracking task. Data from the eye-tracking task have not been analyzed. We collected the KDT because this test is widely used to identify concussions in athletes (24). The KDT results are presented in the Supplementary material.

Following this pre-heading measurement session, participants performed a ball-passing task with their head (“heading task”). One h after the heading task they underwent a second measurement session which was identical to the pre-heading measurement session. The next day, approximately 24 h after the heading task, participants returned for a final measurement session. All three sessions of EEG data were recorded from all 36 participants.

### 2.3 Ball passing task

In this study, we used two versions of a ball-passing task. In one version, participants passed the ball with their feet (“kicking task”). In the other version, participants passed the ball with their head (“heading task”). The heading task served as a model of repetitive sub-concussive impact, and the kicking task served as a control to avoid the potential of confounding due to exercise.

In both versions of the ball-passing task, a soccer ball launcher was placed on a field. A spot was marked 40 feet away from the launcher and in line with the ball trajectory. Soccer balls were inflated to 1.1 kg/cm^2^ consistent with the official guidelines from the International Football Association Board (36). The launcher was calibrated to eject balls at 25 mph (40.2 km/h) using a radar-based speed gun (Velocity Speed Gun, Bushnell Corp.; Overland Park, USA); speed measurements were performed from immediately behind the ball launcher. The launch angle was calibrated for each subject so that the balls were thrown at a comfortable height for the subject to pass back to the experimenter by heading the ball.

Subjects were instructed to pass the ball as accurately as they could to an experimenter standing on a mark 20 feet (6.1 m) from the subject. In the kicking task, participants were instructed to pass the ball with their feet. In the heading task, participants were instructed to pass the ball by bumping it with their head. When the participant verbally confirmed they were ready, they took their position on the mark, and the researchers began launching balls toward the subject at a rate of 1 ball every 30 seconds until the subject headed or kicked the ball 20 times.

Similar soccer-heading paradigms appear in the literature (37, 38), and present only a fraction of the heading a soccer player typically engages in during normal practice and gameplay. Both amateur and collegiate soccer players often perform hundreds or even thousands of headers per season (39, 40). In a soccer game, players might voluntarily head balls moving at 43–53 mph (70–85 km/h), with most opportunities for heading occurring at velocities below 40 mph (65 km/h) (41). Our study, therefore, examines the effects of impacts that are in the normal range of soccer practice and gameplayx.

### 2.4 EEG recordings

In each of the three EEG sessions (pre-impact, 1 h post-impact, and 24 h post-impact), we recorded data under two crossed conditions: with eyes open and closed, and while sitting and standing. A trained EEG technician fit the subject with a cap containing 32 Ag/AgCl electrodes placed according to the international 10–10 system and ensured that electrode impedances remained below 10 k. EEG was recorded in a room with minimal distractions and digitized at 512 Hz using the ANT Neuro eego-sports 32-channel amplifier system (https://www.ant-neuro.com/products/eego_sports). Subjects were instructed to minimize movements and remain in a relaxed but wakeful state. We recorded 8 blocks of EEG, each 2.5 min in duration. EEG was recorded in the following order: sitting with eyes open, sitting with eyes closed, standing with eyes open, standing with eyes closed (followed by another cycle of these four blocks).

### 2.5 EEG analysis

EEG analyses were performed using the mne library and custom-written code in Python.

#### 2.5.1 Preprocessing

EEG data were band-pass filtered from 0.1–50 Hz and re-referenced to the average of all channels. Activity related to eye-movements was then automatically removed using independent component analysis (ICA), rejecting any components that matched either a horizontal or vertical eye-movement template. Finally, we automatically excluded jumps in the raw data by finding the maximum peak-to-trough amplitude in a sliding 0.5 s window. We excluded any segments with a peak-to-trough amplitude greater than 15 standard deviations from the mean, along with 0.5 s before and after those segments.

#### 2.5.2 Feature computation

We then summarized the spectra of each block of EEG data using band-power analyses. We computed these features separately for each experimental block. Features were averaged over each task, yielding four sets of features for each EEG session: eyes open while sitting, eyes closed while sitting, eyes open while standing, and eyes closed while standing.

To derive band-power features, we took the average power within a frequency band separately for each channel. Spectra were computed using Welch's method (NFFT = 512, giving a window length of 1 s, overlapping by 0.5 s). Band-power was computed for the following frequency bands: delta (1–4 Hz), theta (4–8 Hz), alpha (8–13 Hz), beta 1 (13–20 Hz), beta 2 (20–30 Hz), and gamma (30–50 Hz).

These analyses yielded one feature for every combination of task (eyes open/closed), posture (sitting/standing), EEG channel, and frequency band. For example, one feature holds the delta power at channel F3 for the eyes-open-while-sitting block.

### 2.6 Classifier analysis

We used XGBOOST (https://xgboost.readthedocs.io/) classifiers to predict whether a particular EEG session was recorded before the heading task (baseline) or after the heading task (post-impact) on the basis of pre-computed EEG features. Before the EEG features were passed to the XGBOOST models, we performed feature selection within cross-validation based on each feature's F-score. XGBOOST models were trained with 100 estimators, the “gbtree” booster, the “hist” tree method, the binary logistic objective function, the “logloss” evaluation metric, and gamma set to 1. We computed the balanced accuracy of each model using cross-validation (CV) with k = 5. CV was grouped by subject to prevent data leakage (42); the model score is therefore an estimate of the model's performance on a new participant. Each model is trained after specifying a random seed, which determines the random selection of features and observations that are used in training each estimator tree within an XGBOOST model.

Model selection was performed using Tree-Parzen estimators (“hyperopt” method) over 64 search trials, using the tune_sklearn library. Models were selected based on their mean balanced accuracy across CV folds. This process searched for an optimal combination of the following model hyperparameters (using the accompanying distributions): maximum tree depth (uniform distribution of integers between 2 and 8), alpha regularization (log-uniform from 10^−15^–1), lambda regularization (log-uniform from 10^−15^–100), the proportion of observations selected to train each tree (uniform from 0.1–1.0), the proportion of columns selected to train each tree (uniform from 0.2–1.0), the learning rate (log-uniform from 0.01–1.0), and the number of features selected before passing the data to the XGBOOST classifier (uniform from 10 to the number of features in the dataset).

### 2.7 Randomization tests

Model-selection can introduce positive bias into the estimates of model performance by evaluating multiple models and choosing the one with the highest performance. To account for this bias and test whether a model's performance is greater than the performance that would be expected by chance, we used a randomization procedure. First, we fit each model-selection process 100 times with different random seeds, giving a distribution of cross-validated classification performance. Then, we performed another 100 model-searches, shuffling the target labels before each search. This procedure allowed us to test whether the model's true performance was greater than the performance that would be expected by chance (when the labels have been randomly shuffled). We tested for statistical significance by comparing the distributions of performance with true and shuffled labels using two-tailed Welch's *t*-tests of independent samples.

## 3 Results

**Figure 2:**
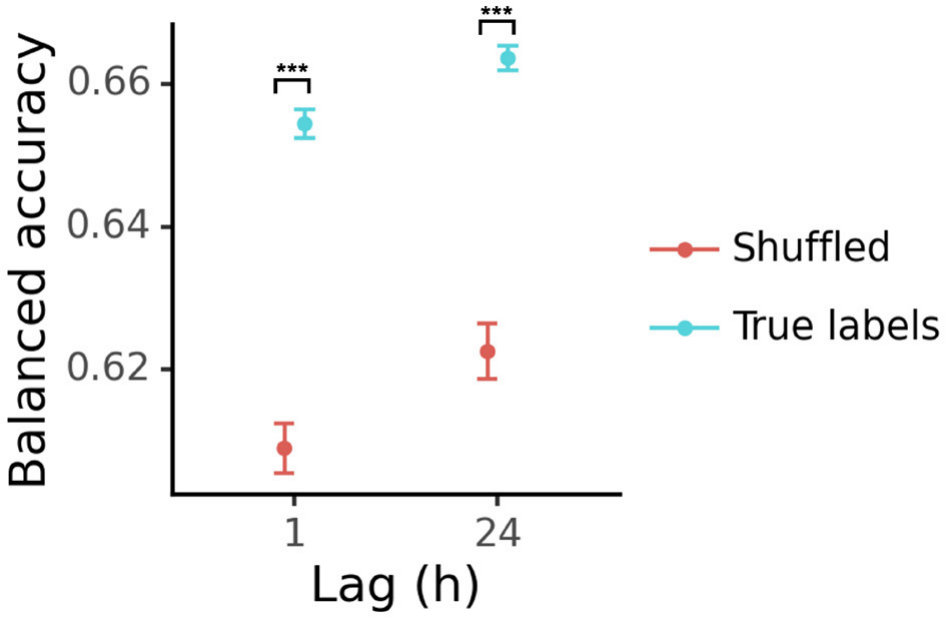
Balanced accuracy of classifiers trained to distinguish between pre-sub-concussive-impact and post-sub-concussive impact EEG features (*N* = 36 at each lag). Each point shows the mean cross-validated balanced accuracy across 100 models trained with different random seeds, and error bars show the standard error. Columns show results for different classifier models (“1 h”: pre-impact vs. 1 h post-impact; “24 h”: pre-impact vs. 24 h post-impact). Blue points show results computed with the true labels, and red points show results computed with shuffled labels (chance performance). ^***^: *p* < 0.001.

In this study, we aimed to test whether resting EEG recordings provide information that can identify whether participants experienced sub-concussive impacts. To address this goal, we trained classifiers to distinguish between pre-impact and post-impact resting EEG. Classifiers were provided with the average power within canonical frequency bands (delta, theta, alpha, beta1, beta2, and gamma) for each of four tasks (sitting with eyes open, sitting with eyes closed, standing with eyes open, and standing with eyes closed). We trained one classifier to distinguish between pre-impact and 1-h post-impact EEG, and another classifier to distinguish between pre-impact and 24-h post-impact EEG. We assessed chance levels of performance using a randomization procedure, and found that the balanced accuracy was significantly higher than would be expected by chance for both the pre- vs. 1-h [*t*_(156.5)_ = 11.33, *p* = 4.2 × 10^−22^] and the pre- vs. 24-h classifiers [*t*_(134.8)_ = 9.43, *p* = 1.6 × 10^−16^; Figure 2].

To determine if the electrophysiological changes following sub-concussive impacts vary over time, we tested whether classifiers trained on EEG recorded at one lag (e.g., 1 h post-heading) could successfully identify EEG recorded at the other recording lag (e.g., 24 h post-heading). To avoid data-leakage, we used cross-validation to assess performance when classifiers were trained and tested on the same lag. Since the pre-impact observations were identical between the 1-h and 24-h comparisons, we only compared model performance on the post-impact sessions. As a consequence of the fact that the classifiers in this analysis are only tested on post-impact observations (which are treated as positive labels), it was not possible to calculate specificity or balanced accuracy. We therefore report the classifier sensitivity.

**Figure 3:**
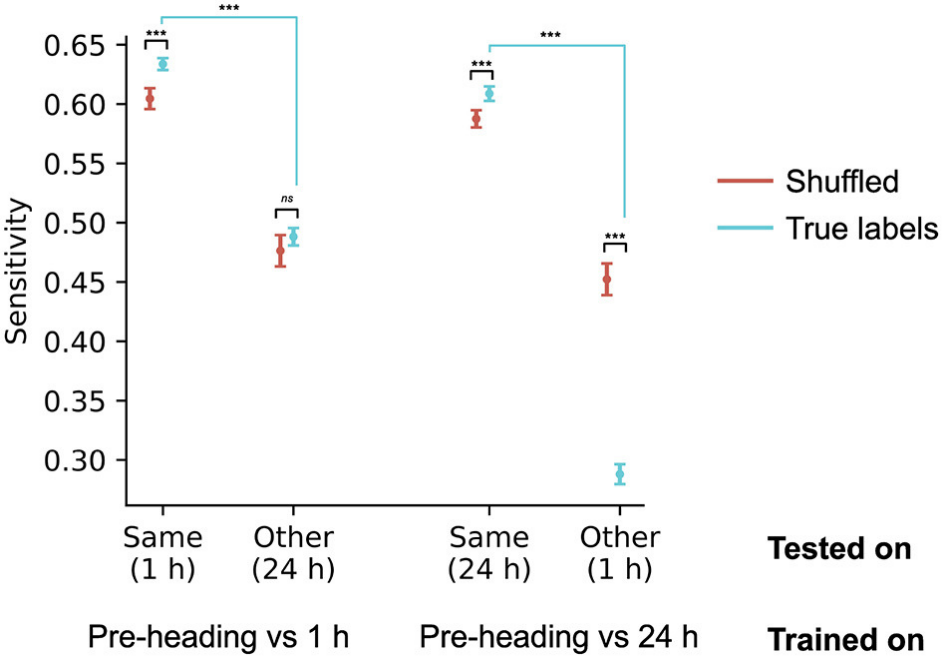
EEG patterns supporting successful classification were different across the two post-impact recording lags (1 h, 24 h; *N* = 36 at each lag). Models were trained to distinguish between pre-impact and one of the two lags, and tested on the same lag used in training, as well as on the other lag. In this analysis, only the positive (post-impact) cases were used, because the negative (pre-impact) cases were identical across the two comparisons; performance is therefore reported using the classifier sensitivity. Each point shows the sensitivity for models trained on one lag (pre-impact vs. 1 h, or pre-impact vs. 24 h), and tested on the same or the other lag. Points show the mean cross-validated sensitivity across 100 models with different random seeds. Error bars show the standard error of the mean. ^***^: *p* < 0.001; *ns*: not significant (*p* > 0.05). Significance annotations with black bars refer to differences between models that used true labels versus models that used shuffled labels (the randomization procedure). Annotations with blue bars refer to differences between models that were tested on different lags (e.g., 1 h vs. 24 h).

When classifiers are trained to distinguish between pre-impact and 1 h post-impact, they do not perform above chance levels at identifying EEG recorded 24 h post-impact (sensitivity = 0.49, *t*_(155.7)_ = 0.78, *p* = 0.44). Performance at the non-trained lag (24 h) is significantly lower than performance at the trained lag (1 h; Figure 3; *t*_(174.4)_ = 16.16, *p* = 1.7 × 10^−36^).

When classifiers are trained to distinguish between pre-impact and 24 h post-impact, they perform significantly lower than chance levels at identifying EEG recorded 1 h post-impact (sensitivity = 0.29, *t*_(166.9)_ = −10.38, *p* = 8.6 × 10^−20^). Performance at the non-trained lag (1 h) is significantly lower than performance at the trained lag (24 h; Figure 3; *t*_(181.5)_=30.67, *p* = 1.0 × 10^−73^). This result suggests that EEG recorded 1 h after sub-concussive impact is more similar to pre-impact EEG than to EEG recorded 24 h after impact. The electrophysiological consequences of sub-concussive impacts, therefore, continue to develop over at least the first 24 h after impact.

These classifiers show above-chance classification performance when distinguishing pre-impact from post-impact EEG at a given delay. To test whether exercise affects model performance, we examined cross-validated balanced accuracy separately within participants who began the study with a kicking task (“kicking group”), and those who began the study without the kicking task (“non-kicking group”).

**Figure 4:**
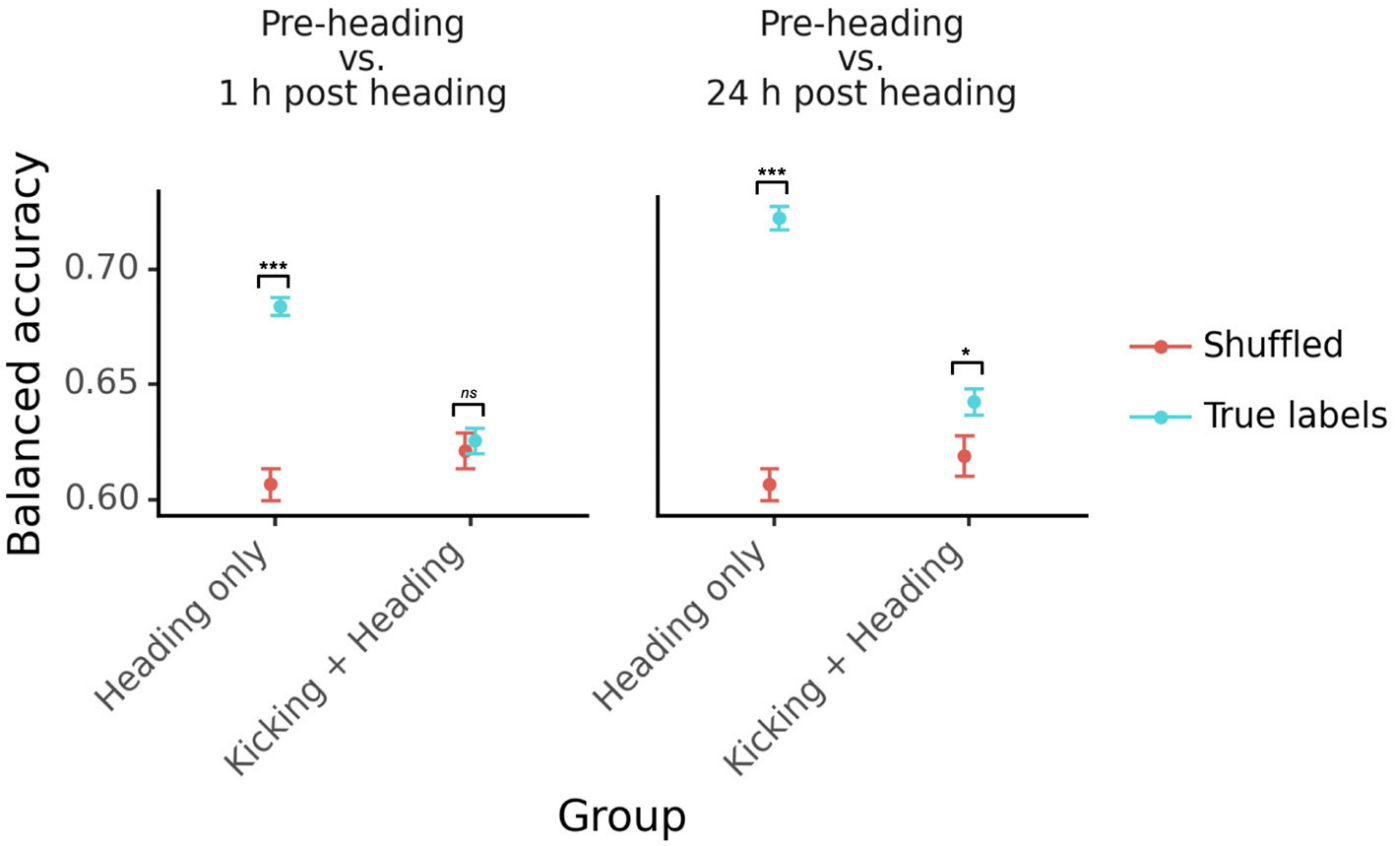
Classifier accuracy plotted separately for participants who performed the kicking task before their baseline EEG session (“Kicking + Heading”) and participants who did not perform any task before their baseline (“Heading Only”). The left panel shows results for the pre-impact vs. 1 h post-impact classifier, and the right panel shows results for the pre-impact vs. 24 h post-impact classifier. Points, error bars, and colors as in Figure 2. ^***^: *p* < 0.001; ^*^: *p* < 0.05; *ns*: not significant (*p* > 0.05).

In the non-kicking group, participants did not exercise before their pre-impact EEG session. As a consequence, above-chance classification performance could reflect the combined brain electrophysiological consequences of sub-concussive impacts and exercise. The non-kicking group shows balanced accuracy that is above the chance levels obtained with shuffled labels, both 1 h after sub-concussive impacts [*t*_(154.3)_ = 9.69, *p* = 1.2 × 10^−17^] and 24 h after sub-concussive impacts [*t*_(180.3)_ = 13.26, *p* = 1.9 × 10^−28^; Figure 4].

In the kicking group, participants exercised before their pre-impact EEG session. Above-chance classification performance in this group, therefore, should primarily reflect the brain electrophysiological consequences of sub-concussive impacts. One h after sub-concussive impacts, the classifiers did not identify post-impact EEG at greater than chance levels [*t*_(179.1)_ = 0.46, *p* = 0.65]. At the later EEG session, 24 h after sub-concussive impacts, the classifiers performed at above-chance levels [*t*_(172.3)_ = 2.25, *p* = 0.026; Figure 4].

These results demonstrate that it is possible to detect the electrophysiological consequences of sub-concussive impacts. We also found that classifiers are influenced by the effects of exercise on EEG signals. This is important because sub-concussive impacts will often occur in the context of physical exercise. Future studies can be designed to accommodate the influence of exercise when training these classifiers.

## 4 Discussion

### 4.1 Overview

There is increasing awareness that sub-concussive impacts have long-term detrimental consequences on the structure and function of the brain. In this study, we developed a preliminary EEG classifier that identifies people who are experiencing early brain electrophysiological effects of these sub-concussive impacts. Consequences of impacts due to soccer ball heading were detectable at 24 h, but not at 1 h, following heading. These results demonstrate that it is possible to use EEG to detect the functional consequences of sub-concussive impacts. Future studies could expand on this work by developing a classifier that can partial out the effects of exercise, to help identify the potential early consequences of repetitive sub-concussive head impacts.

### 4.2 Potential consequences of repetitive sub-concussive impacts during soccer on brain function

Several studies provide evidence that repetitive sub-concussive impacts from soccer ball heading can affect brain function. Many of these studies include testing of cognitive function and a few include imaging measurements of brain function. Although a comprehensive review of all such studies is beyond the scope of this paper, a few examples are included below.

Cognitive dysfunction has been identified amongst active soccer players, with such deficits associating with the frequency of soccer ball heading (43–46). For example, one study of amateur soccer players demonstrated that higher levels of heading during a two-week period was associated with worse performance on tests of psychomotor speed and attention (46). A study of professional soccer players found that the number of headers during one soccer season was inversely related to poorer attention and visual/verbal memory (45). A recently published investigation of working memory found that among amateur soccer players, greater 12-month heading exposure was associated with lower rates of learning among women (but not men) (47). There is controversy about whether cognitive deficits persist after exposure to head impacts has resolved, such as in retired soccer players. Although there are several studies suggesting that deficits persist, other studies did not identify persistent cognitive deficits (48–52).

Imaging studies have identified objective measures of atypical brain function amongst soccer players. A functional near infrared spectroscopy imaging (fNIRS) study of twenty soccer players who each headed a soccer ball 10 times, identified changes in pre-heading to post-heading brain oxygenation and entropy in prefrontal and motor cortex (53). Another study found changes in resting state blink-related oscillations (an EEG-measured neurological response following blinking) amongst a group of ten female soccer players after a single season (34). Furthermore, there was an association between the number of experienced head impacts with increases in delta and beta spectral power post-blinking.

Magnetic resonance spectroscopy (MRS) studies comparing soccer players to healthy controls have demonstrated altered brain regional metabolism. For example, one study found lower N-acetylaspartate/creatinine and higher glutamine and glutamate/creatinine in soccer players (54), perhaps consistent with neuronal injury. A second study found increases in choline (perhaps related to axonal injury, demyelination, or neuroinflammation), and increases in myo-inositol (a marker of glial activation) in soccer players, and showed that these changes in brain metabolism correlated with estimates of the number of recent and lifetime soccer ball headers (55).

An event-related brain potential (ERP) study of soccer players demonstrated that those with repetitive sub-concussive impacts had amplitude reduction in indices of attentional resource allocation and attentional orienting compared with non-contact sport athletes (56).

These studies demonstrate that soccer ball heading likely leads to at least transient alterations in brain function in at least some soccer players. The results from our study demonstrate that the classification of such changes, at 24 h after repetitive soccer ball heading, is feasible with EEG.

### 4.3 Benefits of EEG classifiers over other techniques to identify physiological consequences of repetitive head impacts

EEG classifier models have a number of important benefits over other methods that could be deployed to identify the effects of sub-concussive impacts. First, EEG classifiers do not require a baseline measurement when generating a prediction. This makes an EEG classifier more practical, allowing it to be applied as needed to patients who did not expect to experience head impacts or did not have the opportunity to have baseline testing. Second, classifiers based on resting EEG are not influenced by practice effects, unlike behavioral tests used for detecting TBI. EEG classifiers can therefore be repeatedly administered to monitor brain health over the course of a sports season or a military deployment. Third, EEG classifiers are affordable and portable, compared with detection methods such as functional MRI (57). Furthermore, MRI is impossible in people who are likely to have ferromagnetic shrapnel embedded in their bodies (such as soldiers who have experienced a close-range blast during training).

### 4.4 EEG patterns supporting classification are delayed after sub-concussive impacts

In our study, the electrophysiological consequences of sub-concussive impacts were detectable 24 h after heading a soccer ball, but not 1 h after heading. This delay is consistent with the timing of the inflammatory cascade following TBI. The brain's acute inflammatory response to TBI unfolds over 24–48 h after head trauma (58, 59), and brain physiologic changes continue to develop over the next several days (59–61). Considered alongside our EEG results, this delay suggests that the electrophysiological changes that follow sub-concussive impacts do not reflect the immediate effects of neural damage. Instead, EEG changes may reflect other consequences of TBI, such as neuroinflammation, disruption of the blood-brain barrier, or the cumulative effect of injury cascades that occur immediately after impact.

### 4.5 Limitations

There are several limitations of our study, some of which could be addressed by future research. Our classifier picks up on effects of exercise in addition to the direct effects of sub-concussive impact. There may be sex-specific differences in the effects of soccer ball heading or other repetitive sub-concussive impacts on brain function (47, 62). Our study did not have an adequate sample size to perform meaningful subgroup analyses by sex. It is difficult to account for each research participant's skill with soccer ball heading. Although not quantified, we observed that the soccer players in our study used different techniques for soccer ball heading, including striking the ball with different parts of their head and with differing amounts of neck movements. These differences in heading technique may have affected how much impact soccer ball heading had on brain function. Finally, since there is not yet a gold-standard measurement technique for detecting changes in brain function due to repetitive sub-concussive events, we were forced to calculate our classification accuracy assuming that all research participants had changes in brain function following the soccer ball heading task. However, some participants might not have actually experienced such changes. In that case, the classifier's inability to differentiate post-heading and pre-heading EEG data in some of the subjects would be consistent with accurate classification. Thus, our results demonstrate that the aftereffects of sub-concussive events can be detected with our EEG classifier, but the exact accuracy is unknown.

## Data Availability

The datasets presented in this article are not readily available because participants in this study consented to having their data shared only with Mayo Clinic research staff, sponsor research staff, Mayo Clinic staff involved in participant care, the IRB, and government agencies that oversee or review research. Data will be provided upon request to those approved by consent. Requests to access the datasets should be directed to geoff.brookshire@sparkneuro.com.
