# Supplementary material for: Measuring electrophysiological changes induced by sub-concussive impacts due to soccer ball heading
Source: Front Neurol. 2025 Mar 6;16:1500796. doi: 10.3389/fneur.2025.1500796 (PMC11922700; doi:10.3389/fneur.2025.1500796)
Supplement: Supplementary file 1 [file Data_Sheet_1.pdf]

Supplementary Material

1 KING-DEVICK TEST

1.1 Methods

The King Devick Test (KDT) is a visual test in which participants read aloud sequences of numbers Galetta et al. (2015). This test is widely used to identify concussions in athletes . In the KDT, participants read aloud from three cards with rows of variably spaced numbers, as quickly and accurately as possible. The score on the test is determined as the sum of the time taken to read the three cards. The test is determined to be positive (indicating that the participant is concussed) when the total time taken to perform the test is longer than the time taken by the participant to complete their baseline KDT. We administered the KDT in each EEG session. We used the pre-impact KDT as the necessary baseline value, yielding two measurements for each subject: at 1 h and 24 h after subconcussive impacts.

1.2 Results

One hour after subconcussive impacts, we obtained positive KDT results in 43% (16/37) of participants. At the later session, 24 h after subconcussive impacts, we obtained positive KDT results in 14% (5/37) of participants.

1.3 Discussion

Results demonstrate that EEG detects subclinical effects of soccer ball heading at 24 hours. In other words, although only 14% of participants had abnormal KDT at 24 hours after heading, the EEG classifier accurately identified EEG recordings collected at that time point. Since the task-free EEG recording seems to be more sensitive to post-heading functional consequences in the brain and since it does not require a pre-injury measurement for comparison, we propose that a classifier based on task-free resting EEG could be particularly useful.

REFERENCES

Galetta, K. M., Liu, M., Leong, D. F., Ventura, R. E., Galetta, S. L., and Balcer, L. J. (2015). The King-Devick test of rapid number naming for concussion detection: meta-analysis and systematic review of the literature. *Concussion* 1, CNC8. doi:10.2217/cnc.15.8
